# Axial length association with corneoscleral sagittal height and scleral asymmetry

**DOI:** 10.1111/opo.13402

**Published:** 2024-10-03

**Authors:** Elena Martínez‐Plaza, Alberto López‐de la Rosa, Ainhoa Molina‐Martín, Laurent Bataille, David P. Piñero

**Affiliations:** ^1^ Group of Optics and Visual Perception, Department of Optics, Pharmacology and Anatomy University of Alicante Alicante Spain; ^2^ University of Valladolid Valladolid Spain; ^3^ Department of Theoretical Physics, Atomic and Optics University of Valladolid Valladolid Spain; ^4^ Instituto de Oftalmobiología Aplicada (IOBA) University of Valladolid Valladolid Spain; ^5^ Visitrain S.L. Science Park of the University of Alicante Alicante Spain; ^6^ Department of Ophthalmology Vithas Medimar International Hospital Alicante Spain

**Keywords:** axial length, cornea, corneoscleral profile, eye surface profiler, sagittal height, sclera

## Abstract

**Purpose:**

To determine how corneoscleral geometry changes with axial length and to assess the usefulness of including the sagittal configuration of the anterior segment when predicting the axial length.

**Methods:**

An observational study was performed including 96 healthy subjects (96 eyes). Axial length was calculated from optical biometry (IOL Master 500). Corneal curvature and scleral sagittal height parameters at 13, 14 and 15 mm were obtained automatically using corneoscleral topography (eye surface profiler; ESP). In addition, corneal and scleral sagittal heights at numerous locations (21 radii: 0–10 mm from the corneal apex at 12 angles: 0–330°) were calculated using the raw height data extracted from the ESP. The relationships between axial length and the study parameters were analysed using Pearson correlation analysis. The equations for the prediction of axial length were obtained by fitting multiple linear regression models.

**Results:**

The temporal‐nasal scleral asymmetry at 13‐, 14‐ and 15‐mm chord lengths was significantly correlated with axial length (*r* ^2^ ≤ 0.26; *p* < 0.001). Significant inverse correlations were found between the temporal scleral sagittal height and axial length (*r* ^2^ ≤ 0.28; *p* ≤ 0.02). The nasal scleral sagittal height was not associated with axial length. Three significant multiple linear regression models were fitted based on spherical equivalent, corneal radius and scleral asymmetry at 13 (*r* ^2^ = 0.79; *p* < 0.001), 14 (*r* ^2^ = 0.80; *p* < 0.001) and 15 (*r* ^2^ = 0.80; *p* < 0.001) mm chord lengths.

**Conclusions:**

Larger ocular globes show reduced temporal‐nasal scleral asymmetry, mainly due to the lower sagittal height of the temporal sclera. Thus, the geometry of the temporal scleral may be a factor of interest during myopia progression.

**Key points**

- The temporal‐nasal sagittal height difference in the scleral area was lower in larger ocular globes.
- Whilst the nasal scleral area remained stable, the temporal area decreased in terms of sagittal height as the axial length increased.
- Adding scleral asymmetry to the refractive error and corneal curvature values does not appear to improve accuracy when predicting the axial length of the eye.

## INTRODUCTION

The refractive state of the eye results from the combined contribution of various ocular biometric parameters, including corneal power, anterior chamber depth, vitreous chamber depth, crystalline lens power and axial length.^1^, ^2^ In emmetropic eyes, the contribution of all these components is balanced, so that with relaxed accommodation, rays from optical infinity converge at the retina. However, when this harmony is disrupted, refractive errors such as myopia, hyperopia or astigmatism arise. Specifically, myopia often arises due to an elongated axial length, so that with relaxed accommodation, rays from optical infinity converge in front of the retina.^3^ In recent years, myopia has become a major public health concern due to a substantial increase in prevalence,^4^ alongside the costs associated with its management.^5^, ^6^ This has increased scientific interest in understanding the ocular implications associated with myopia development and axial growth.

Axial elongation has been associated with changes in scleral shape. Some findings, such as tissue thinning, have been observed in both the anterior and posterior sclera.^7^, ^8^, ^9^, ^10^ The anterior sclera is markedly more accessible in vivo than the posterior region. Additionally, the anterior sclera exhibits temporal‐nasal asymmetry,^11^ although the magnitude is reduced in eyes with longer axial length.^12^ However, it is unknown whether the contribution of each region changes with axial length elongation (for instance whether the temporal sagittal height decreases, the nasal height increases or both change in opposite directions), as well as the effect at different chord lengths (i.e., 13, 14 and 15 mm). This knowledge would be of great interest to some clinical areas, such as designing and fitting scleral contact lenses.

In addition, the use of predictive formulas of axial length based on parameters that are easily collectable for most clinicians, may help to define new strategies for the prevention, detection and control of myopia. For example, a previous study combining refractive error and corneal radius has demonstrated good predictive capability.^13^ However, this prediction could be improved by adding additional parameters. In a preliminary study,^14^ our research group observed that some corneoscleral parameters, along with the refractive error, could predict axial length successfully. Nonetheless, this study was limited by the small sample size. Therefore, the objectives of the present study were firstly to increase knowledge about how each region of the corneosclera changes with axial length in healthy individuals, and secondly to assess the accuracy in predicting axial length from the sagittal configuration of the anterior segment.

## METHODS

The present work is an observational study approved by the Ethics Committee of the University of Alicante (Alicante, Spain), adhering to the tenets of the Declaration of Helsinki. The study was conducted at the optometry clinic of the University of Alicante and written informed consent was obtained from all participants.

### Sample

The inclusion criteria comprised adult participants with healthy eyes. Exclusion criteria were the presence or history of any ocular anomaly or pathology, prior ocular surgery, the application of topical medications, pregnancy or wearing rigid gas‐permeable contact lenses. Volunteers who wore soft contact lenses were asked to discontinue their use for 3 days before the measurements. Subjects meeting the inclusion criteria were included consecutively. Both eyes of all participants were examined, although one eye was selected at random for statistical analysis.

### Clinical evaluation

Slit‐lamp biomicroscopy was conducted to identify anomalies of the ocular surface. Manifest refraction and best‐corrected distance visual acuity were assessed using the logarithm of the minimum angle of resolution (logMAR).

### Optical biometry

Axial length was obtained after four consecutive measurements per eye using an IOL Master 500 (Carl Zeiss Meditec AG, zeiss.com/), with all measurements being obtained by the same operator (EMP). The mean of the four values was used. These measurements were obtained in a closed dark room during the same study visit.

### Corneoscleral assessment

The eye surface profiler (ESP) topography system (Eaglet Eye b.v., eaglet‐eye.com) was used to obtain the corneoscleral surface geometry. The examination adhered to the evaluation protocol described by Iskander et al.^15^ Initially, a topical application of a mixture containing Aquawet eye drops and fluorescein sodium (BioGlo Fluorescein Sodium Ophthalmic Strips USP; hubrx.com) was administered to stain the ocular surface. Participants were then positioned within the device and instructed to fixate on the target, whilst the operator gently retracted their eyelids without applying pressure to the globe. Three repeated corneoscleral topographic profiles were performed per eye by the same experienced clinician (EMP) and the mean value calculated.

**FIGURE 1:**
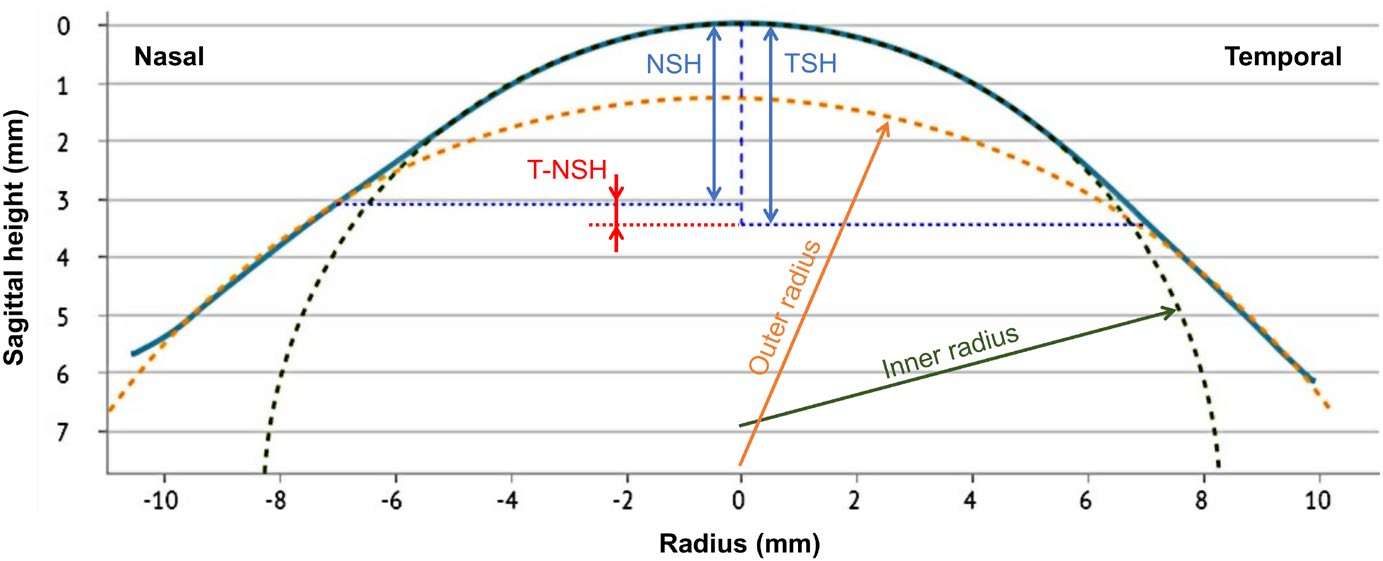
Corneoscleral section, modified from the ESP software, representing the inner and outer radii and nasal (NSH), temporal (TSH) and temporal‐nasal (T‐NSH) sagittal heights at 14 mm chord length.

The following parameters obtained using the ESP were collected at each evaluation: simulated keratometry in the steep and flat meridians, mean keratometry (average of simulated keratometry in the steep and flat meridians), inner radius (radius of the corneal sphere), outer radius (radius of the scleral sphere) and sagittal height measurements for 13‐, 14‐ and 15‐mm chord lengths. The sagittal height measurements included average sagittal height, difference between the temporal and nasal sagittal heights, minimum and maximum sagittal height and minimum and maximum sagittal height of the largest orthogonal difference. Figure 1 represents a corneoscleral section describing the main study parameters.

Additionally, the raw height data provided by the ESP was extracted and managed as reported previously.^16^ Briefly, the sagittal height location provided in Cartesian format (*X*‐, *Y*‐ coordinates) was transformed into polar format (radius, angle). Sagittal height from 21 radii (corneal apex to 10 mm in 0.5 mm steps) at 12 angles (ranging from 0° to 330° in 30° steps) were considered. Data from the left eyes were horizontally flipped to align the temporal and nasal areas with those of the right eyes. Finally, parameters with discrepancies >0.2 mm between measurements (a reasonable value according to the device precision for sagittal height^17^) were excluded to avoid the influence of outliers (e.g., noise of the marginal area), resulting in the exclusion of the subject for that specific parameter.

### Statistical analysis

The statistical analysis was performed using R version 4.2.3 (r‐project.org). The sample size was calculated to detect in a Pearson correlation analysis a coefficient of determination of 0.1873, as reported previously in a preliminary study,^14^ with a significance level of 5% and a statistical power of 90%. Based on these criteria, 52 subjects were required. However, in a previous study with the same methodology, sufficient data were collected for analysing the parameters with a 14 mm of chord length from 58% of eyes.^16^ Therefore, to assure sufficient information for this chord length, a 42% potential loss of data was considered. Therefore, the final sample size was at least 90 subjects. Nonetheless, when acceptable data were not obtained from at least 52 subjects, the parameter was not analysed statistically.

The relationship between the axial length and each of the study parameters (keratometry values, sagittal heights, etc.) was evaluated using the Pearson correlation analysis. To minimise the likelihood of a Type I error arising from the multiple independent hypothesis tests being conducted, the false‐discovery rate (FDR) method was employed to adjust the *p*‐values.^18^ *p*‐Values ≤0.05 were considered statistically significant. Additionally, the ability of the refractive, corneal and scleral parameters to predict the axial length was analysed by fitting multiple linear regression models. Three different models were fitted, considering the sagittal height parameters of the sclera at three different chords (13‐, 14‐ and 15‐mm). The backward elimination method was applied using a *p*‐value threshold of 0.05 for variable selection. The assumptions of normality, lack of multicollinearity, linearity, homoscedasticity and lack of outliers were checked by analysing the residual plots, using the Kolmogorov–Smirnov test and calculating the variance inflation factor.

## RESULTS

### Study population

Ninety‐six eyes (50 right and 46 left eyes) from 96 participants (24 males and 72 females) with a mean age of 31.7 (13.7) years were involved in the present study. Table 1 shows descriptive data of the sample population.

**TABLE 1:** Descriptive data of the sample.

Parameter	*n*	Mean (SD)
Visual acuity (logMAR)	96	−0.08 (0.06)
Sphere (dioptres)	96	−1.72 (2.42)
Cylinder (dioptres)	96	−0.48 (0.54)
Spherical equivalent (dioptres)	96	−1.96 (2.48)

### Relationship between axial length and corneoscleral geometry

**FIGURE 2:**
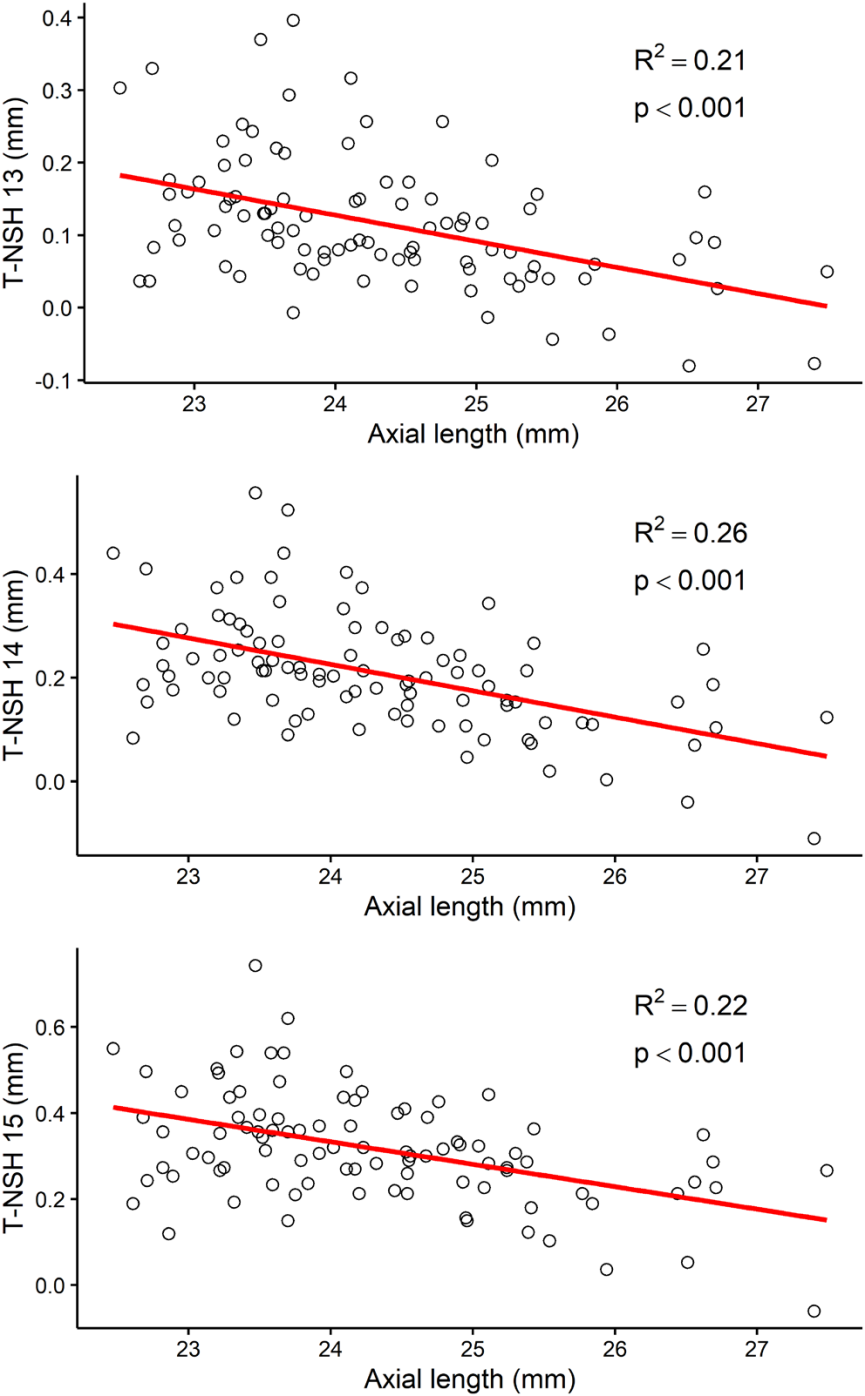
Correlation analysis showing the relationship between the axial length and temporal‐nasal sagittal height at 13 mm (top), 14 mm (middle) and 15 mm (bottom) chord lengths. T‐NSH, difference between temporal and nasal sagittal heights.

The corneoscleral characteristics provided by the ESP and its association with axial length are given in Table 2. The temporal‐nasal scleral asymmetry at 13‐, 14‐ and 15‐mm chord lengths were the most highly correlated parameters with axial length (0.21 ≥ *r* ^2^ ≤ 0.26), as shown in Figure 2.

**TABLE 2:** Mean data and correlation analysis between axial length and corneoscleral parameters provided by the eye surface profiler.

Parameter	*n*	Mean (SD)	Pearson's *r* (95% CI)	*r* ^2^	Adjusted *p*‐value
AL (mm)	96	24.31 (1.13)	NA	NA	NA
SimKs (mm)	96	7.83 (0.27)	0.34 (0.15/0.51)	0.12	**0.005**
SimKf (mm)	96	8.18 (0.29)	0.30 (0.10/0.47)	0.09	**0.01**
MeanK (mm)	96	8.00 (0.27)	0.33 (0.14/0.50)	0.11	**0.006**
Inner radius (mm)	96	8.40 (0.28)	0.23 (0.03/0.41)	0.05	0.07
Outer radius (mm)	96	12.79 (0.58)	0.29 (0.10/0.47)	0.08	**0.01**
ASH 13 (mm)	96	2.86 (0.13)	−0.16 (−0.35/0.04)	0.03	0.16
T‐NSH 13 (mm)	96	0.12 (0.09)	−0.45 (−0.6/−0.28)	0.21	**<0.001**
MinSH 13 (mm)	96	2.76 (0.15)	−0.04 (−0.24/0.16)	0.00	0.70
MaxSH 13 (mm)	96	2.93 (0.13)	−0.21 (−0.4/−0.01)	0.05	0.08
MinSH90° 13 (mm)	69	2.79 (0.14)	−0.16 (−0.38/0.08)	0.03	0.25
MaxSH90° 13 (mm)	69	2.92 (0.14)	−0.28 (−0.48/−0.04)	0.08	0.06
ASH 14 (mm)	96	3.24 (0.15)	−0.18 (−0.36/0.02)	0.03	0.14
T‐NSH 14 (mm)	96	0.21 (0.11)	−0.51 (−0.65/−0.35)	0.26	**<0.001**
MinSH 14 (mm)	96	3.11 (0.16)	−0.06 (−0.25/0.15)	0.00	0.63
MaxSH 14 (mm)	96	3.31 (0.15)	−0.20 (−0.38/0.001)	0.04	0.10
MinSH90° 14 (mm)	46	3.15 (0.15)	−0.19 (−0.46/0.10)	NA	NA
MaxSH90° 14 (mm)	46	3.30 (0.15)	−0.31 (−0.55/−0.03)	NA	NA
ASH 15 (mm)	96	3.61 (0.16)	−0.17 (−0.36/0.03)	0.03	0.14
T‐NSH 15 (mm)	94	0.32 (0.13)	−0.46 (−0.61/−0.29)	0.22	**<0.001**
MinSH 15 (mm)	96	3.48 (0.17)	−0.08 (−0.27/0.12)	0.01	0.51
MaxSH 15 (mm)	96	3.70 (0.17)	−0.20 (−0.38/0.006)	0.04	0.10
MinSH90° 15 (mm)	20	3.51 (0.15)	−0.28 (−0.65/0.18)	NA	NA
MaxSH90° 15 (mm)	20	3.69 (0.15)	−0.45 (−0.75/−0.01)	NA	NA

Abbreviations: AL, axial length; ASH, average sagittal height; CI, confidence interval; MaxSH, maximum sagittal height; MaxSH90°, maximum sagittal height at 90 degrees; MeanK, mean simulated keratometry; MinSH minimum sagittal height; MinSH90°, minimum sagittal height at 90 degrees; NA, not applicable; SD, standard deviation; SimKs, simulated keratometry in the steep meridian; SimKf, simulated keratometry in the flat meridian; T‐NSH, difference between temporal and nasal sagittal heights.

*Note:* Bold values indicate significant adjusted *p*‐values.

**FIGURE 3:**
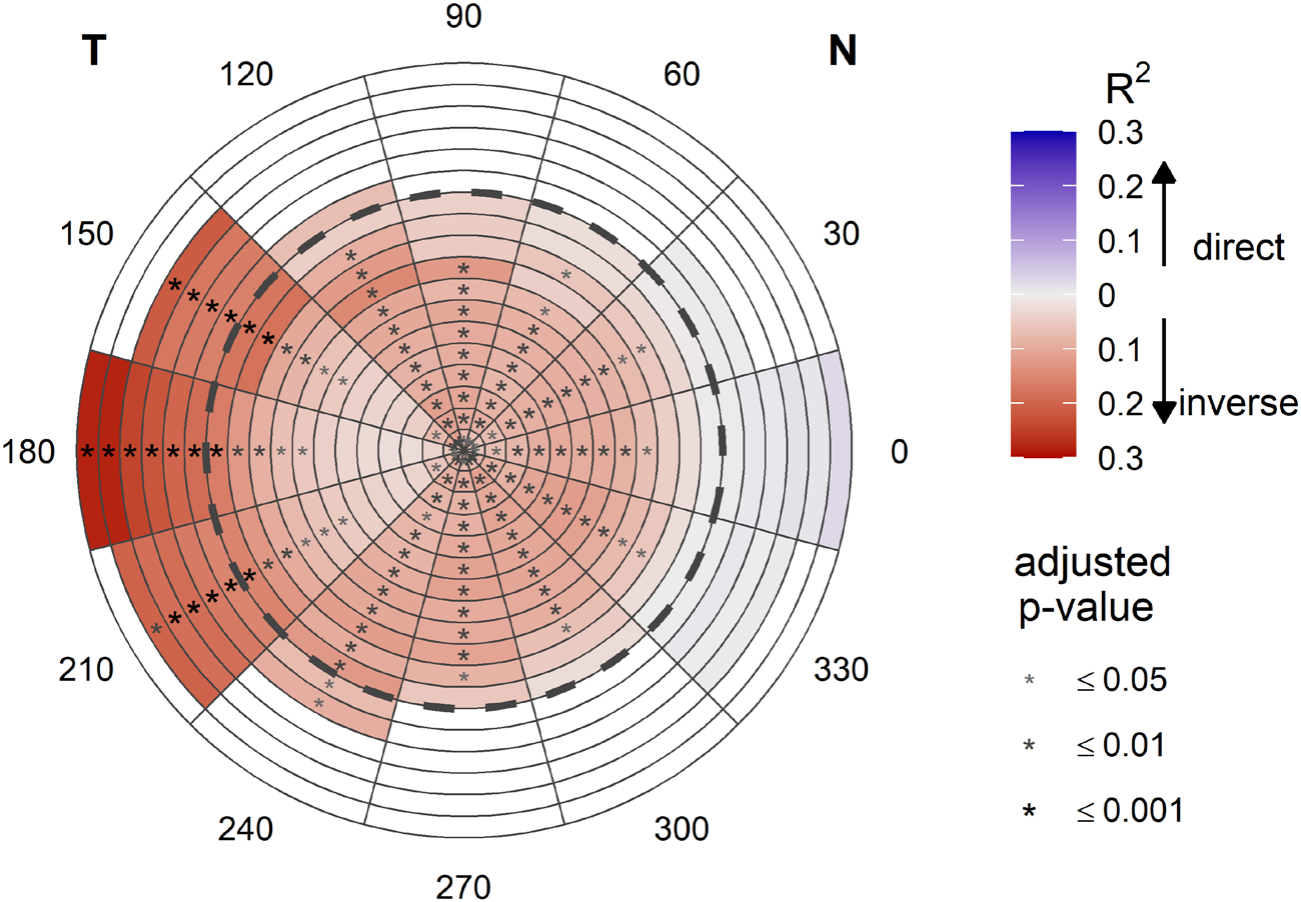
Colour map representation of the relationship between the axial length and corneoscleral sagittal height. The centre of the map represents the corneal apex, and the dashed line divides the cornea (≤12 mm diameter) from the sclera (>12 mm). Cold and warm colours represent direct and inverse correlations, respectively. Parameters with a sample size lower than 52 eyes are presented in white. N, nasal; T, temporal.

The relationship between the axial length and corneal and scleral sagittal heights is represented in Figure 3. At the cornea, inverse relationships were found between the axial length and numerous sagittal heights around the 360° of the cornea. At the sclera, inverse relationships were found between the axial length and sagittal heights in the temporal sclera (Table 3); however, the sagittal heights in the nasal sclera were not significantly associated with axial length.

**TABLE 3:** Mean data and statistically significant correlations between axial length and scleral sagittal height parameters.

Radius	Angle	*n*	Mean (SD)	Pearson's *r* (95% CI)	*r* ^2^	Adjusted *p*‐value
13 mm	150°	93	2.97 (0.12)	−0.40 (−0.56/−0.21)	0.16	<0.001
	180°	95	3.01 (0.14)	−0.43 (−0.58/−0.25)	0.19	<0.001
	210°	96	3.06 (0.15)	−0.39 (−0.55/−0.21)	0.15	<0.001
	240°	74	3.04 (0.16)	−0.27 (−0.47/−0.04)	0.07	0.03
14 mm	150°	87	3.41 (0.14)	−0.41 (−0.57/−0.22)	0.17	<0.001
	180°	94	3.50 (0.17)	−0.45 (−0.60/−0.27)	0.20	<0.001
	210°	93	3.54 (0.19)	−0.41 (−0.57/−0.23)	0.17	<0.001
	240°	57	3.50 (0.19)	−0.30 (−0.52/−0.05)	0.09	0.03
15 mm	150°	82	3.86 (0.17)	−0.42 (−0.58/−0.22)	0.17	<0.001
	180°	86	3.99 (0.20)	−0.47 (−0.62/−0.29)	0.22	<0.001
	210°	89	4.00 (0.22)	−0.43 (−0.59/0.25)	0.19	<0.001
16 mm	150°	71	4.33 (0.19)	−0.46 (−0.63/−0.25)	0.21	<0.001
	180°	84	4.49 (0.23)	−0.49 (−0.64/−0.31)	0.24	<0.001
	210°	78	4.50 (0.25)	−0.43 (−0.60/−0.23)	0.18	<0.001
17 mm	180°	78	4.97 (0.25)	−0.53 (−0.67/−0.35)	0.28	<0.001
	210°	64	5.00 (0.28)	−0.45 (−0.63/−0.23)	0.20	0.002
18 mm	180°	65	5.48 (0.28)	−0.53 (−0.69/−0.33)	0.28	<0.001

Abbreviations: CI, confidence interval; SD, standard deviation.

### Predictability of axial length

Three significant multiple linear regression models were fitted based on spherical equivalent, corneal radius and scleral parameters at 13 (*r* ^2^ = 0.79; *F*_3,92_ = 120.4; *p* < 0.001), 14 (*r* ^2^ = 0.80; *F*_3,92_ = 129.4; *p* < 0.001) and 15 (*r* ^2^ = 0.80; *F*_3,90_ = 122.8; *p* < 0.001) mm chord lengths. Tables 4, 5, 6 provide model coefficients for the prediction of axial length.

**TABLE 4:** Coefficients of the multiple linear regression models for the prediction of axial length with refractive, corneal and 13 mm scleral parameters.

Predictors	Coefficient (95% CI)	Standardised coefficient (95% CI)	*t*	*p*‐Value
Intercept	10.21 (7.04/13.37)		6.41	<0.001
Spherical equivalent (D)	−0.35 (−0.40/−0.31)	−0.87 (−0.99/−0.76)	−14.77	<0.001
SimKs (mm)	1.73 (1.33/2.13)	0.46 (0.35/0.57)	8.61	<0.001
T‐NSH 13 (mm)	−1.45 (−2.76/−0.15)	−0.13 (−0.25/−0.01)	−2.21	0.03

Abbreviations: CI, confidence interval; SimKs, simulated keratometry in the steep meridian; T‐NSH, difference between temporal and nasal sagittal heights.

**TABLE 5:** Multiple linear regression model coefficients for the prediction of axial length with refractive, corneal and 14 mm scleral parameters.

Predictors	Coefficient (95% CI)	Standardised coefficient (95% CI)	*t*	*p*‐Value
Intercept	10.81 (7.70/13.92)		6.90	<0.001
Spherical equivalent (D)	−0.34 (−0.39/−0.30)	−0.85 (−0.96/−0.74)	−14.77	<0.001
SimKs (mm)	1.68 (1.29/2.07)	0.45 (0.34/0.55)	8.56	<0.001
T‐NSH 14 (mm)	−1.67 (−2.69/−0.66)	−0.19 (−0.30/−0.07)	−3.28	0.001

Abbreviations: CI, confidence interval; SimKs, simulated keratometry in the steep meridian; T‐NSH, difference between temporal and nasal sagittal heights.

**TABLE 6:** Multiple linear regression model coefficients for the prediction of axial length with refractive, corneal and 15 mm scleral parameters.

Predictors	Coefficient (95% CI)	Standardised coefficient (95% CI)	*t*	*p*‐Value
Intercept	10.74 (7.57/13.92)		6.73	<0.001
Spherical equivalent (D)	−0.35 (−0.40/−0.30)	−0.88 (−0.99/−0.76)	−14.88	<0.001
SimKs (mm)	1.69 (1.29/2.09)	0.45 (0.34/0.56)	8.42	<0.001
T‐NSH 15 (mm)	−1.21 (−2.12/−0.30)	−0.16 (−0.27/−0.04)	−2.65	0.01

Abbreviation: CI, confidence interval; SimKs, simulated keratometry in the steep meridian; T‐NSH, difference between temporal and nasal sagittal heights.

## DISCUSSION

The axial length of the globe has been associated with changes in scleral geometry. Specifically, it appears that the sagittal height of the sclera in the horizontal meridian (temporal‐nasal) is more symmetrical in larger eyes.^11^, ^12^, ^19^ However, little is known about how each specific region of the sclera is modified as the axial length of the eyeball increases. Thus, this study aimed to assess how corneal and scleral geometry varies with axial length, and to determine whether the sagittal configuration of the anterior segment provides useful information for estimating axial length.

The sclera is known to show greater sagittal height in the temporal versus the nasal area.^14^, ^19^, ^20^, ^21^ In addition, both previous studies^11^, ^14^, ^19^ and the present investigation observed that temporal‐nasal scleral asymmetry is inversely correlated with axial length. This means that larger globes show smaller sagittal height differences between the temporal and nasal scleral regions. This study also analysed the sagittal height of numerous locations which indicated that the main contributor that diminishes the asymmetry is the temporal sclera. Specifically, the temporal sclera is inversely correlated with axial length, whereas the nasal sclera shows no significant correlation (Figure 3). These results are in line with Lee et al.,^22^ showing that the temporal scleral radius of curvature increases (i.e., flattens) with greater axial length. On the other hand, Niyazmand et al.^19^ found an inverse correlation between the spherical equivalent and nasal sagittal height (i.e., greater nasal sagittal height in more myopic eyes), although no such correlation was observed for the temporal area. These differences may be due to the fact that refractive error is influenced by factors independent of the external geometry of the eyeball (e.g., lens position, power or curvature), which could introduce noise into the results. Additionally, refraction may vary with the meridian being examined. Therefore, results considering the axial length may be more consistent. In summary, as axial length increases, the sagittal height of the temporal area decreases, resulting in lower temporal‐nasal scleral asymmetry.

Additionally, it was noted in the temporal scleral area that the correlation between sagittal height and axial length was stronger as the location became more peripheral (Table 3, Figure 3). The limit of the area studied (data were analysed up to an 18 mm diameter) corresponds approximately to the insertions of the medial and lateral rectus muscles (being 5.7 ± 0.8 mm and 6.8 ± 0.7 mm from the limbus, respectively).^23^ In fact, these muscle insertions may be responsible for the differences found between the temporal and nasal scleral areas, as previously proposed.^14^, ^19^ In particular, the possible difference in tension applied by the muscles due to the difference in insertion location, in conjunction with weakening of the biomechanical properties of the scleral tissue in myopic eyes (i.e., more elastic,^24^ less rigid^25^ and thinner^7^, ^8^, ^9^, ^10^), may somehow explain these differences. However, this hypothesis may be clarified by further studies analysing these specific aspects.

The present study evaluated the predictive potential of various anterior segment and refractive parameters for estimating axial length. This could be useful for predicting this parameter in clinical centres lacking an optical biometer; for example, during contact lens fitting for myopia control where a corneoscleral topography may be available. The current results indicate that the temporal‐nasal asymmetry of the sclera at 13, 14 or 15 mm, in conjunction with the spherical equivalent and the simulated keratometry values, could be useful to determine the axial length, since they account for about 80% of the variability. Other authors have also found a similar coefficient of determination without including scleral asymmetry or considering the subject's age.^13^, ^26^ Therefore, it appears that including scleral asymmetry does not increase predictive capacity; however, its statistical significance in the model confirms its relationship with axial length.

The present study had some limitations. Eyelids were retracted while avoiding ocular globe compression to increase the exposed area. However, there were missing values, mainly in the superior and inferior sclera. Additionally, fixating the internal target of the device may lead to proximal accommodation, which may be considered as a confounding factor. However, it should be noted that this is part of the evaluation protocol and is required for proper alignment when using the ESP device. Another limitation was the absence of cycloplegia when performing subjective refraction, which was used to calculate the spherical equivalent for the predictive formulas. However, this study did not include children, whose subjective refraction without cycloplegic agents is more prone to be influenced by accommodation.^27^ Further, most eye care practitioners perform subjective refraction without cycloplegia, making the results more representative of daily clinical practice. Finally, the age range of the subjects was large, which may have introduced bias due to the possible effect of age on scleral geometry. However, a previous study did not find any association between scleral asymmetry and age.^12^

In conclusion, larger ocular globes show less temporal‐nasal scleral asymmetry, primarily due to the geometry of the temporal sclera. Specifically, the sagittal height of the temporal sclera was lower in the larger eyes, whilst the nasal height remained stable. Therefore, temporal scleral geometry may be a factor of interest to consider during myopia progression.

## AUTHOR CONTRIBUTIONS

**Elena Martínez‐Plaza:** Conceptualization (supporting); data curation (equal); formal analysis (equal); investigation (equal); methodology (equal); writing—original draft (lead); writing—review and editing (equal). **Alberto López‐de la Rosa:** Conceptualization (supporting); data curation (equal); formal analysis (equal); methodology (equal); software (lead); writing—original draft (lead); writing—review and editing (equal). **Ainhoa Molina‐Martín:** Investigation (equal); methodology (equal); writing—review and editing (equal). **Laurent Bataille:** Methodology (equal); writing—review and editing (equal). **David P. Piñero:** Conceptualization (lead); funding acquisition (lead); investigation (equal); methodology (equal); project administration (lead); resources (lead); supervision (lead); writing—review and editing (equal).

## FUNDING INFORMATION

This research received no external funding. E.M.‐P. has been supported by European Union‐NextGenerationEU.

## CONFLICT OF INTEREST STATEMENT

No author has a financial or proprietary interest in any of the materials or methods mentioned. The authors have no financial disclosures to declare.
